# Investigation of Heterochromatin Protein 1 Function in the Malaria Parasite Plasmodium falciparum Using a Conditional Domain Deletion and Swapping Approach

**DOI:** 10.1128/mSphere.01220-20

**Published:** 2021-02-03

**Authors:** Hai T. N. Bui, Armin Passecker, Nicolas M. B. Brancucci, Till S. Voss

**Affiliations:** a Department of Medical Parasitology and Infection Biology, Swiss Tropical and Public Health Institute, Basel, Switzerland; b University of Basel, Basel, Switzerland; University at Buffalo

**Keywords:** CRISPR/Cas9, DiCre, HP1, *Plasmodium falciparum*, epigenetics, heterochromatin, malaria

## Abstract

The human malaria parasite Plasmodium falciparum encodes a single ortholog of heterochromatin protein 1 (PfHP1) that plays a crucial role in the epigenetic regulation of various survival-related processes. PfHP1 is essential for parasite proliferation and the heritable silencing of genes linked to antigenic variation, host cell invasion, and sexual conversion. Here, we employed CRISPR/Cas9-mediated genome editing combined with the DiCre/loxP system to investigate how the PfHP1 chromodomain (CD), hinge domain, and chromoshadow domain (CSD) contribute to overall PfHP1 function. We show that the 76 C-terminal residues are responsible for targeting PfHP1 to the nucleus. Furthermore, we reveal that each of the three functional domains of PfHP1 are required for heterochromatin formation, gene silencing, and mitotic parasite proliferation. Finally, we discovered that the hinge domain and CSD of HP1 are functionally conserved between P. falciparum and P. berghei, a related malaria parasite infecting rodents. In summary, our study provides new insights into PfHP1 function and offers a tool for further studies on epigenetic regulation and life cycle decision in malaria parasites.

**IMPORTANCE** Malaria is caused by unicellular *Plasmodium* species parasites that repeatedly invade and replicate inside red blood cells. Some blood-stage parasites exit the cell cycle and differentiate into gametocytes that are essential for malaria transmission via the mosquito vector. Epigenetic control mechanisms allow the parasites to alter the expression of surface antigens and to balance the switch between parasite multiplication and gametocyte production. These processes are crucial to establish chronic infection and optimize parasite transmission. Here, we performed a mutational analysis of heterochromatin protein 1 (HP1) in P. falciparum. We demonstrate that all three domains of this protein are indispensable for the proper function of HP1 in parasite multiplication, heterochromatin formation, and gene silencing. Moreover, expression of chimeric proteins revealed the functional conservation of HP1 proteins between different *Plasmodium* species. These results provide new insight into the function and evolution of HP1 as an essential epigenetic regulator of parasite survival.

## Abstract (precis)

Malaria is caused by unicellular *Plasmodium* species parasites that repeatedly invade and replicate inside red blood cells. Some blood-stage parasites exit the cell cycle and differentiate into gametocytes that are essential for malaria transmission via the mosquito vector.

## INTRODUCTION

Heterochromatin protein 1 (HP1) was initially described in Drosophila melanogaster as a nonhistone chromosomal protein associated with heterochromatin and responsible for variegated gene expression (1, 2). By binding to the repressive tri-methylation mark on lysine 9 of histone 3 (H3K9me3) (3, 4), HP1 facilitates the formation and spreading of heterochromatin. Chromatin-bound HP1 serves as a platform for the recruitment of downstream chromatin modifiers, including H3K9-specific histone SU(VAR)3-9-like lysine methyltransferases (HKMT) that methylate H3K9 on neighboring nucleosomes and thus facilitate the binding of further HP1 proteins (5, 6). The self-propagation of HP1 results in the regional spreading of heterochromatin, thereby promoting silencing of heterochromatin-associated genes (7–10).

HP1 is a small protein and well conserved among eukaryotes (11). Orthologs have been identified in a broad range of unicellular and multicellular organisms (7, 11), including the evolutionarily divergent protozoan parasites of the genus *Plasmodium*, the causative agents of malaria (12, 13). Schizosaccharomyces pombe contains two members of the HP1 protein family, namely, Switching 6 (Swi6) and chromodomain-containing protein 2 (Chp2); three variants (HP1α, HP1β, HP1γ) are encoded in the mammalian genome, and D. melanogaster possesses five HP1 variants (HP1a to -e) (8, 11). HP1 proteins consist of three distinct functional domains: a conserved chromodomain (CD) at the N terminus, which binds H3K9me3 (3, 4); a conserved chromoshadow domain (CSD) at the C terminus, which mediates HP1 dimerization and interaction with other chromatin modifiers (5, 14–17); and a variable hinge region separating the CD and CSD and shown to interact with histone H1, DNA, or RNA (18–21).

In mammals, chromatin localization of HP1 proteins has been shown to be variant specific. While mammalian HP1α and HP1β are found primarily in pericentromeric heterochromatin, HP1γ is found in both heterochromatic and euchromatic regions (22–25). The requirements for targeting HP1 to heterochromatin show interspecies differences. In fission yeast, the CD was shown to direct Swi6 to heterochromatin (26), whereas in mice, this function additionally requires RNA binding by the hinge domain (19). In D. melanogaster HP1, both the 95 N-terminal residues (encompassing the CD) and C-terminal residues 95 to 206 (containing the hinge domain and CSD) target HP1 to pericentromeric heterochromatin (27, 28). Similarly, HP1 domains play different roles in targeting HP1 to the nucleus in different species. In D. melanogaster, the 54 C-terminal residues of the protein (amino acids 152 to 206) are required for importing HP1 to the nucleus (27). In S. pombe, however, the hinge region of Swi6 plays a dominant role in directing Swi6 to the nucleus. Additionally, the Swi6 C terminus acts as a second, albeit weaker, nucleus-targeting domain (26).

Malaria parasites possess only a single HP1 ortholog, which binds primarily to chromosomal regions containing members of multigene families encoding variant surface antigens (12, 13, 29, 30). Unlike its orthologs in other eukaryotes (31–34), however, HP1 is absent from pericentromeric regions in malaria parasites and plays no apparent role in the formation and maintenance of centromere structure and function (12, 29, 30). In Plasmodium falciparum, the species causing the most severe form of malaria in humans, PfHP1 is associated with the subtelomeric regions of all chromosomes and with some chromosome-internal islands (12, 30), where H3K9me3 is also enriched (35, 36). These heterochromatic regions contain over 400 protein-coding genes, most of which belong to gene families encoding exported virulence proteins and variant surface antigens, including the *var* gene family (12, 30). The *var* gene family consists of 60 paralogs encoding antigenically and functionally distinct variants of P. falciparum erythrocyte membrane protein 1 (PfEMP1) that are displayed on the surfaces of infected red blood cells (iRBCs) (37–40). The interaction of PfEMP1 with receptors on endothelial cells or uninfected RBCs results in cellular adherence and sequestration of iRBCs in the microvasculature, which is a major cause of severe malaria symptoms (41–43). In addition, antigenic variation and sequence diversity of PfEMP1 variants contribute significantly to immune evasion and hence to the establishment of chronic infection (41). Antigenic variation of PfEMP1 is based on switches in the mutually exclusive transcription of *var* genes (44). At any given time, only a single *var* gene is expressed, while the remaining *var* gene family members are transcriptionally silenced (singular gene choice) (41, 44–46). *var* gene silencing is linked to the presence of H3K9me3/PfHP1 at the promoter and coding region (12, 13, 35, 36, 47–49). The single active *var* gene, however, is instead enriched in H3K9ac and H3K4me3 in the upstream regulatory region (47). How the switch from the silenced to the active state is mediated is not known, but perinuclear locus reposition is linked to this process (50–55).

In addition to silencing virulence gene families, HP1 silences the gene encoding AP2-G, the master transcriptional regulator of gametocytogenesis in malaria parasites (29, 30, 49, 56, 57). AP2-G is a member of the ApiAP2 family of putative transcription factors of apicomplexan parasites (59). The *ap2-g* gene is located in a chromosome-internal H3K9me3/HP1-demarcated heterochromatic island (12, 29, 30, 35, 36). Work on P. falciparum has shown that the PfHP1-dependent silencing of *pfap2-g* prevents sexual conversion and secures continuous parasite proliferation cycles. Removal of PfHP1 from the *pfap2-g* locus triggers the expression of PfAP2-G and irreversible sexual conversion (49). Parasites that express AP2-G give rise to sexually committed progeny that exit the mitotic cell cycle and differentiate into female or male gametocytes (49, 56, 57, 60–64). Gametocytes are the only parasite stages capable of infecting the mosquito vector and as such are essential for malaria transmission. In P. falciparum, activation of *pfap2-g* transcription depends on nuclear protein gametocyte development 1 (GDV1), which binds to and expels PfHP1 from the *pfap2-g* locus (60). Interestingly, even though *ap2-g* is associated with HP1 in all *Plasmodium* species (30), activation of this locus in P. berghei and other *Plasmodium* species infecting rodents is independent of GDV1, as they lack a GDV1 ortholog (60, 65, 66).

By analyzing a conditional PfHP1 knockdown mutant, we previously demonstrated that PfHP1 is essential for gene silencing and mitotic parasite proliferation (49). In that study, PfHP1-depleted parasites completed the current intraerythrocytic multiplication cycle and gave rise to ring-stage progeny, in which the transcription of *var* genes and other heterochromatic gene families was highly augmented. Approximately 50% of this progeny underwent gametocytogenesis (25-fold increase compared to the level in the isogenic control population) due to transcriptional derepression of the *pfap2-g* locus in the previous cell cycle. The other half of the PfHP1-depleted progeny represented asexual parasites that failed to enter schizogony due to defective genome replication (49). To interrogate the function of PfHP1 in more detail, we conducted a functional analysis of PfHP1 domains by combining CRISPR/Cas9-mediated genome editing and the DiCre/loxP system for conditional mutagenesis (67–69). We show that the 76 C-terminal residues encompassing the CSD (amino acids 191 to 266) are responsible for targeting PfHP1 to the nucleus. We further demonstrate that all three PfHP1 domains are required for heterochromatin formation, parasite proliferation, and *pfap2-g* silencing and are therefore indispensable for proper PfHP1 function. Finally, we reveal that the HP1 hinge domain and CSD are functionally conserved between P. falciparum and the rodent malaria parasite P. berghei.

## RESULTS

### Investigation of the roles of PfHP1 domains in PfHP1 localization.

**FIG 1:**
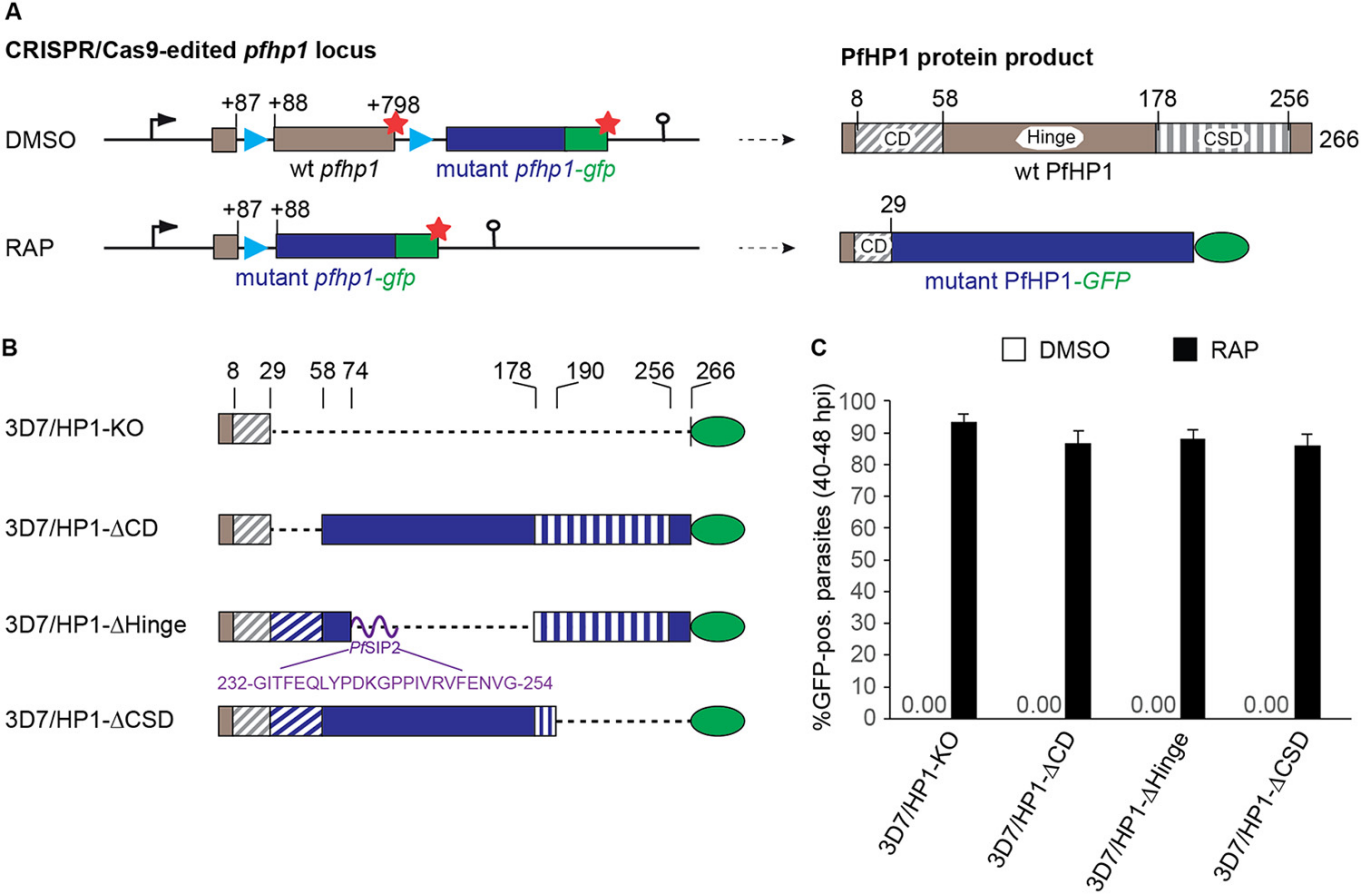
Generation of DiCre-inducible PfHP1 truncation mutants. (A) Schematics of the CRISPR/Cas9-edited *pfhp1* locus (left) and corresponding PfHP1 protein products (right) before (DMSO) and after (RAP) rapamycin-induced DiCre-dependent excision of the floxed wild-type (wt) *pfhp1* locus. Blue arrowheads indicate the position of *sera2* intron:loxP elements. Red stars indicate stop codons. Brown and blue boxes represent the wild-type and the replacing mutant sequences *pfhp1/*PfHP1, respectively. Green boxes represent the *gfp/*GFP sequence. The CD, hinge domain, and CSD within wild-type PfHP1 are indicated. Numbers in the gene and protein schematics refer to nucleotide and amino acid positions, respectively. (B) Diagrams showing the organization of the PfHP1 truncation mutants expressed in the 3D7/HP1-KO, 3D7/HP1-ΔCD, 3D7/HP1-ΔHinge, and 3D7/HP1-ΔCSD lines after RAP treatment. Dashed lines represent corresponding deletions in the mutant PfHP1 protein sequences. Brown and blue colors represent the remaining wild-type PfHP1 N terminus and the replacing mutant protein sequences, respectively. The CD and CSD are indicated by diagonal and vertical dashed stripes, respectively. The purple curved line represents the short PfSIP2-derived linker polypeptide between the CD and CSD in the PfHP1-ΔHinge mutant. The amino acid sequence of this linker and its position within the PfSIP2 protein are indicated. Numbers on top indicate amino acid positions within wild-type PfHP1. (C) Proportion of GFP-positive parasites observed 40 h after treatment with RAP or DMSO (control). Values represent the means of results from three independent biological replicates (error bars indicate SD). For each sample, >200 iRBCs were counted. pos., positive.

To begin studying the functional contribution of individual PfHP1 domains, we engineered parasites that allow for the conditional expression of PfHP1 mutants based on the DiCre/loxP system (67, 68) using CRISPR/Cas9-based gene editing. In these parasites, the floxed endogenous *pfhp1* gene is excised upon rapamycin (RAP)-induced activation of the dimerizable Cre (DiCre) recombinase and replaced with a recodonized *pfhp1* gene encoding a mutated PfHP1 protein carrying a C-terminal green fluorescent protein (GFP) tag (Fig. 1A and see Fig. S1 in the supplemental material). We successfully used this approach recently to study the role of PfHP1 phosphorylation in regulating PfHP1 function (69). Here, we generated four such conditional PfHP1 mutant cell lines called 3D7/HP1-KO, 3D7/HP1-ΔCD, 3D7/HP1-ΔHinge, and 3D7/HP1-ΔCSD, where amino acid residues 30 to 266 (full-length PfHP1), 30 to 58 (CD), 75 to 177 (hinge region), and 191 to 266 (CSD), respectively, are deleted upon RAP treatment (Fig. 1B). In the HP1-ΔHinge mutant, we replaced the hinge domain (102 amino acids) with a short linker peptide (22 amino acids) derived from the ApiAP2 transcription factor PfSIP2, where this peptide connects the two adjacent AP2 DNA-binding domains (70). The CRISPR/Cas9-based gene editing strategy used to generate these parasite lines is explained in detail in Materials and Methods and in Fig. S1. PCR of parasite genomic DNA (gDNA) was performed to confirm the correct integration of the recodonized mutant *pfhp1*-*gfp* gene variants directly downstream of the endogenous *pfhp1* gene as well as the successful DiCre-mediated excision of the floxed endogenous *pfhp1* gene upon RAP treatment in all cell lines (Fig. S1).

10.1128/mSphere.01220-20.2 FIG S1 CRISPR/Cas9-based gene-editing strategy to generate DiCre-inducible PfHP1 truncation and PfHP1-PbHP1 hybrid mutants. (A, top) Schematic map of the *pfhp1* locus (PF3D7_1220900) in 3D7/N31DC parasites. 3D7/N31DC parasites have previously been obtained by inserting a 103-bp *sera2* intron:loxP element (67) (light-blue triangle) into the 5′ end of the *pfhp1* gene in 3D7/1G5DiCre parasites (68) using CRISPR/Cas9-based gene editing (69). The nucleotide positions of the sgt_*pfhp*1-3′ single guide RNA (sgRNA) target sequence is indicated (chromosome 12 coordinates). (Center) Schematic maps of a pD-PfHP1 donor plasmid cotransfected with the pBF-gC-guide250 CRISPR/Cas9 transfection vector (69). The pD-PfHP1 donor plasmids contain an assembly of the 103-bp sera2 intron:loxP element (light-blue triangle) and a mutated *pfhp1* sequence (dark blue) fused to *gfp* (green) flanked by 5′ and 3′ homology regions (HR) (sandstone, black) for homology-directed repair. The pBF-gC-guide250 plasmid contains expression cassettes for SpCas9 (dark gray), the sgRNA (purple), and the *bsd-yfcu*pD-hyb-PbHinge fusion selection marker (light gray to brown). (Bottom) Schematic maps of the modified *pfhp1* loci after CRISPR/Cas9-based gene editing in 3D7/HP1-KO, 3D7/HP1-ΔCD, 3D7/HP1-ΔHinge, 3D7/HP1-ΔCSD, 3D7/HP1-hyb-PbHinge, and 3D7/HP1-hyb-PbCSD are shown. Light-blue triangles represent *sera2* intron:loxP elements, and red stars represent stop codons. Brown and blue boxes represent the wild type and the replacing mutant *pfhp1* sequences, respectively. Numbers refer to the nucleotide position within the *pfhp1* coding sequence. The black arrowheads indicate the binding sites of the F119 and R157 PCR primers used to confirm correct gene editing of the *pfhp1* locus and efficient DiCre-mediated excision upon RAP treatment. (B) PCR confirmation of correct editing of the *pfhp1* locus and efficient excision of the floxed *pfhp1* gene after RAP treatment. The schematic on top shows the *pfhp1* locus before (DMSO) and after RAP treatment in the 3D7/HP1-ΔCSD parasite line as an example. PCRs were performed on gDNA of DMSO-treated (−) and RAP-treated (+) 3D7/HP1-Control (69), 3D7/HP1-hyb-PbHinge, 3D7/HP1-hyb-PbCSD, 3D7/HP1-KO, 3D7/HP1-ΔCD, 3D7/HP1-ΔHinge, and 3D7/HP1-ΔCSD cell lines using the primer combination F119 and R157. The length of PCR fragments amplified from the correctly edited *pfhp1* locus before (−) and after (+) excision upon RAP treatment from the various PfHP1 mutant parasite lines and the 3D7/N31DC mother line are indicated at the bottom of the figure. The correct excision of floxed DNA after RAP treatment (+) results in a decreased fragment size compared to that generated under the DMSO-treated (−) control condition. Download FIG S1, TIF file, 2.4 MB. Copyright © 2021 Bui et al. 2021 Bui et al. This content is distributed under the terms of the Creative Commons Attribution 4.0 International license.

**FIG 2:**
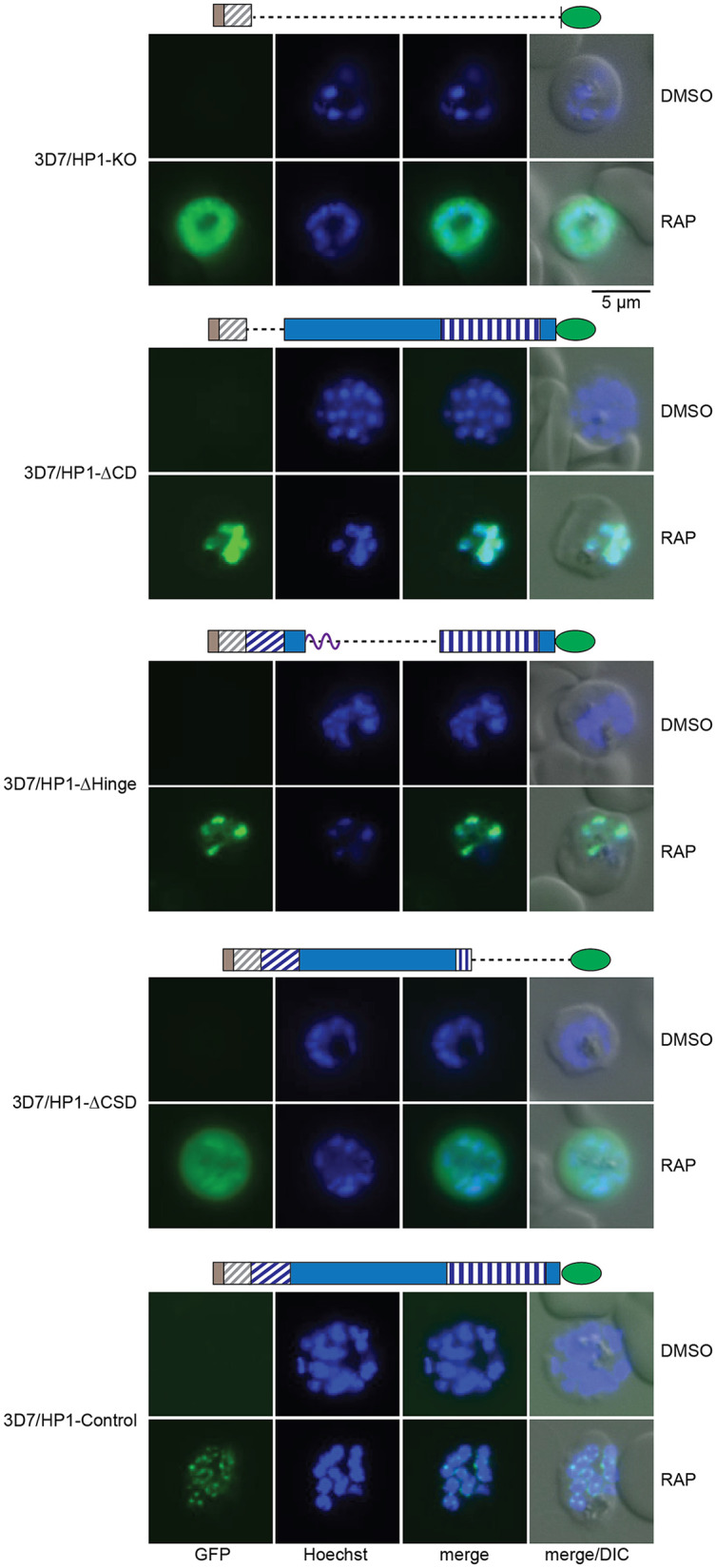
Subcellular localization of PfHP1 truncation mutants. Representative live-cell fluorescence images showing the localization of the PfHP1-GFP fusions in DMSO- and RAP-treated 3D7/HP1-KO, 3D7/HP1-ΔCD, 3D7/HP1-ΔHinge, 3D7/HP1-ΔCSD, and 3D7/HP1-Control lines at late schizont stage (LS [40 to 48 hpi]; generation 1, 40 h after RAP treatment). Nuclei were stained with Hoechst dye. DIC, differential interference contrast. Scale bar, 5 μm.

Scoring GFP-positive parasites by live-cell fluorescence imaging at 40 to 48 h postinvasion (hpi) (generation 1, 40 h after RAP treatment) confirmed the highly efficient excision of the endogenous *pfhp1* gene and expression of PfHP1-GFP mutant proteins upon RAP treatment in all four transgenic parasite lines (Fig. 1C). In contrast, and as expected, parasites in the dimethyl sulfoxide (DMSO)-treated control populations did not express the GFP-tagged PfHP1 mutant proteins (Fig. 1C). With regard to subcellular localization, we observed that the PfHP1ΔCD-GFP and PfHP1ΔHinge-GFP fusion proteins localized to the nucleus (Fig. 2). The PfHP1ΔCSD-GFP protein exhibited reduced nuclear staining, and a substantial fraction localized to the cytoplasm (Fig. 2). Thus, the C-terminal polypeptide encompassing the CSD (amino acids 191 to 266) is required for efficient targeting of PfHP1 to the nucleus. Consistent with this observation, the small fusion protein expressed in the 3D7/HP1-KO line, which contains only the first 29 amino acids of PfHP1 fused to GFP, localized to the cytoplasm (Fig. 2B). The NucPred (71) and PSORTII (https://psort.hgc.jp/form2.html) algorithms identified putative canonical nuclear localization signals (NLSs) in each of the three PfHP1 domains: a KKKK motif in the CD (amino acids 17 to 20), a PRRK motif in the hinge domain (amino acids 100 to 103), and a RRKK motif in the CSD (amino acids 201 to 204). Our fluorescence microscopy-based analysis suggest that the NLS consensus motif in the CSD may function as a major nuclear import element. The predicted NLS in the hinge domain may mediate some level of nuclear targeting, whereas the KKKK motif in the CD seems to lack such activity. Importantly, however, even though the PfHP1ΔCD-GFP and PfHP1ΔHinge-GFP domain deletion mutants were efficiently imported into the nucleus, they failed to assemble into heterochromatin domains but localized diffusely throughout the nucleoplasm. Similarly, the small fraction of nucleus-localized PfHP1ΔCSD-GFP proteins appeared to colocalize with the entire Hoechst dye-stained area of the nucleus (Fig. 2). Typical perinuclear heterochromatic foci could be observed only in control parasites expressing full-length PfHP1-GFP after RAP treatment (3D7/HP1-Control) (Fig. 2).

In summary, these data show that the 76 C-terminal amino acids comprising the CSD are responsible for the efficient targeting of PfHP1 to the nucleus. Inside the nucleus, the CD, hinge domain, and CSD of PfHP1 are all required for the formation of perinuclear heterochromatin.

### Each PfHP1 domain is essential for asexual proliferation and gene silencing in blood-stage parasites.

**FIG 3:**
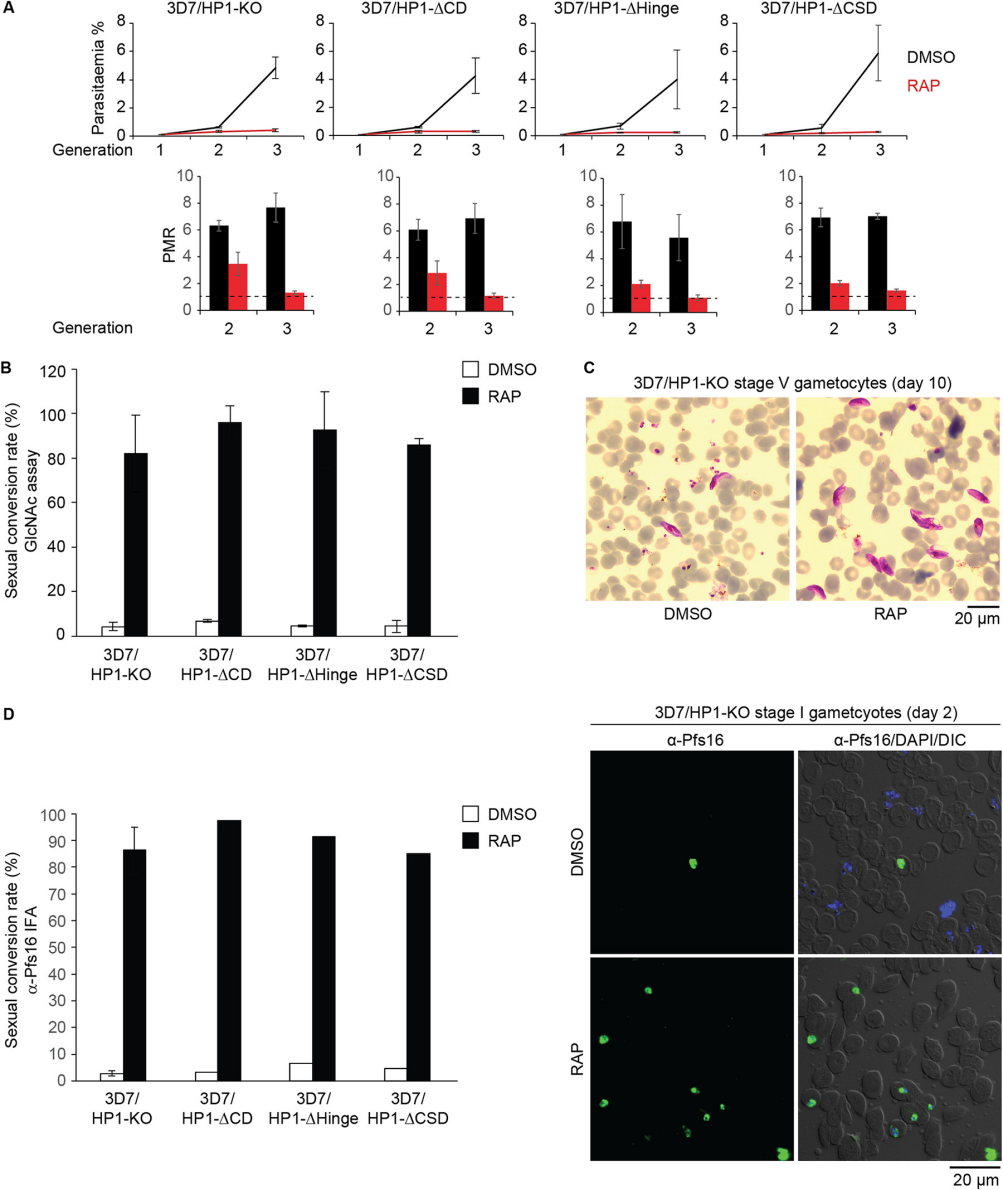
Phenotypes of PfHP1 truncation mutants. (A, top) Growth curves of the DMSO- and RAP-treated PfHP1 truncation mutants over three consecutive generations. (Bottom) Parasite multiplication rates (PMRs) reflect the fold increase in parasitemia observed in generations 2 and 3. Values are the means of results from three biological replicates (error bars indicate SD). For each sample, >3,000 RBCs were counted. (B) Sexual conversion rates of PfHP1 truncation mutants in DMSO- and RAP-treated parasites, assessed by inspection of Giemsa-stained blood smears of GlcNAc-treated cultures on day 6 of gametocytogenesis. Results are the means of results from at least three replicates (error bars indicate SD). For each sample, >3,000 RBCs were counted. (C) Representative overview images from Giemsa-stained blood smears showing stage V gametocytes (day 10 of gametocytogenesis) obtained from DMSO- and RAP-treated 3D7/HP1-KO parasites. Scale bar, 20 μm. (D) Sexual conversion rates of PfHP1 truncation mutants in DMSO- and RAP-treated parasites, assessed by anti-Pfs16 IFAs performed on stage I gametocytes on day 2 of gametocytogenesis. The result from the 3D7/HP1-KO line represents the mean of results from four replicates (error bar indicates SD). All other values derive from a single experiment. For each sample, >200 iRBCs were counted. Representative overview images of anti-Pfs16 IFAs used to quantify stage I gametocytes in DMSO- and RAP-treated populations of the 3D7/HP1-KO line are shown on the right. Scale bar, 20 μm.

We next investigated the importance of each of the three PfHP1 domains for PfHP1 function. First, we compared levels of parasite proliferation in the four PfHP1 truncation mutants grown under control conditions (DMSO) and after RAP treatment. To this end, parasite cultures (0.1% ring-stage parasitemia) were split (generation 1); one half was treated with 100 nM RAP, and the other half was treated with the DMSO solvent (control). For each cell line, the paired populations were monitored over three generations by assessing the parasitemia in the second and third generations by inspection of Giemsa dye-stained blood smears. As shown in Fig. 3A, the parasitemia of all DMSO-treated control populations increased 5- to 7-fold during each of the two multiplication cycles, as expected. The RAP-treated 3D7/HP1-KO line showed a 2-fold-reduced parasite multiplication rate (PMR) after the first replication cycle, and the progeny failed to replicate further (Fig. 3A). This result is entirely consistent with the results of our previous study using a conditional PfHP1-GFP-DD knockdown line, where PfHP1-depleted parasites multiplied at a 2-fold-reduced rate during the first cycle and were unable to proliferate further (49). Interestingly, we observed the same phenotype for all three PfHP1 domain deletion mutants (Fig. 3A), which demonstrates that each individual PfHP1 domain (CD, hinge domain, CSD) is required for the proper function of PfHP1 in controlling the mitotic proliferation of asexual blood-stage parasites.

We previously showed that next to the cell cycle-arrested trophozoites, the proliferation-defective progeny of the conditional PfHP1-GFP-DD knockdown mutant contained a large proportion of early-stage gametocytes due to defective silencing of *pfap2-g* (49). Here, we examined a possible role of individual PfHP1 domains in controlling gene silencing by determining sexual conversion rates (SCRs) as a proxy for *pfap2-g* silencing in DMSO- and RAP-treated parasites for all PfHP1 truncation mutants. To quantify SCRs, the ring-stage progeny of DMSO- and RAP-treated parasites (generation 2, day 1 of gametocytogenesis) were cultured in medium containing *N-*acetyl-d-glucosamine (GlcNAc) to eliminate asexual parasites (72, 73), and gametocytemia was determined after 6 days of GlcNAc treatment (stage III gametocytes). The DMSO-treated control populations of all conditional PfHP1 truncation mutants and the 3D7/HP1-Control line consistently displayed default SCRs of 4 to 7% (Fig. 3B). As expected from the results previously obtained with the conditional PfHP1-GFP-DD knockdown line (49), the SCR of RAP-treated 3D7/HP1-KO parasites was massively increased, reaching 82.1% (±17.3 standard deviations [SD]), which is substantially higher than the average SCR of 52% reported upon knocking down PfHP1-GFP-DD expression (Fig. 3B). Furthermore, as with PfHP1-GFP-DD knockdown gametocytes, PfHP1 null gametocytes of the 3D7/HP1-KO line differentiated into stage V gametocytes in the absence of PfHP1 expression (Fig. 3C). Strikingly, we found that all three RAP-treated 3D7/HP1-ΔCD, 3D7/HP1-ΔHinge, and 3D7/HP1-ΔCSD PfHP1 domain deletion mutants displayed similarly high SCRs of 85 to 95% (Fig. 3B). These results were independently confirmed with immunofluorescence assays (IFAs) quantifying SCRs based on the expression of the gametocyte-specific marker Pfs16 (74) in the progeny of DMSO- and RAP-treated parasites (40 to 48 hpi, day 2 of gametocytogenesis) (Fig. 3D). Together, these findings demonstrate that all three PfHP1 domains are essential for the function of PfHP1 in mediating gene silencing and reinforce the central role for PfHP1 in suppressing sexual conversion.

### The functions of the HP1 hinge and chromoshadow domains are conserved between P. falciparum and P. berghei.

It has been shown that replacement of the CD of S. pombe Swi6 with the CD of the mouse HP1 protein M31 retains Swi6 function in sporulation, normal zygote ascus formation, and mitotic stability. CSD substitution, however, did not restore Swi6 function in these processes (26). Here, we tested the functional conservation between the HP1 orthologs of P. falciparum and P. berghei, the most widely used mouse malaria model parasite. We were interested primarily in this question because P. berghei parasites lack the GDV1 protein that is required for PfHP1 eviction from the *pfap2-g* locus and subsequent sexual conversion in P. falciparum (60, 65).

**FIG 4:**
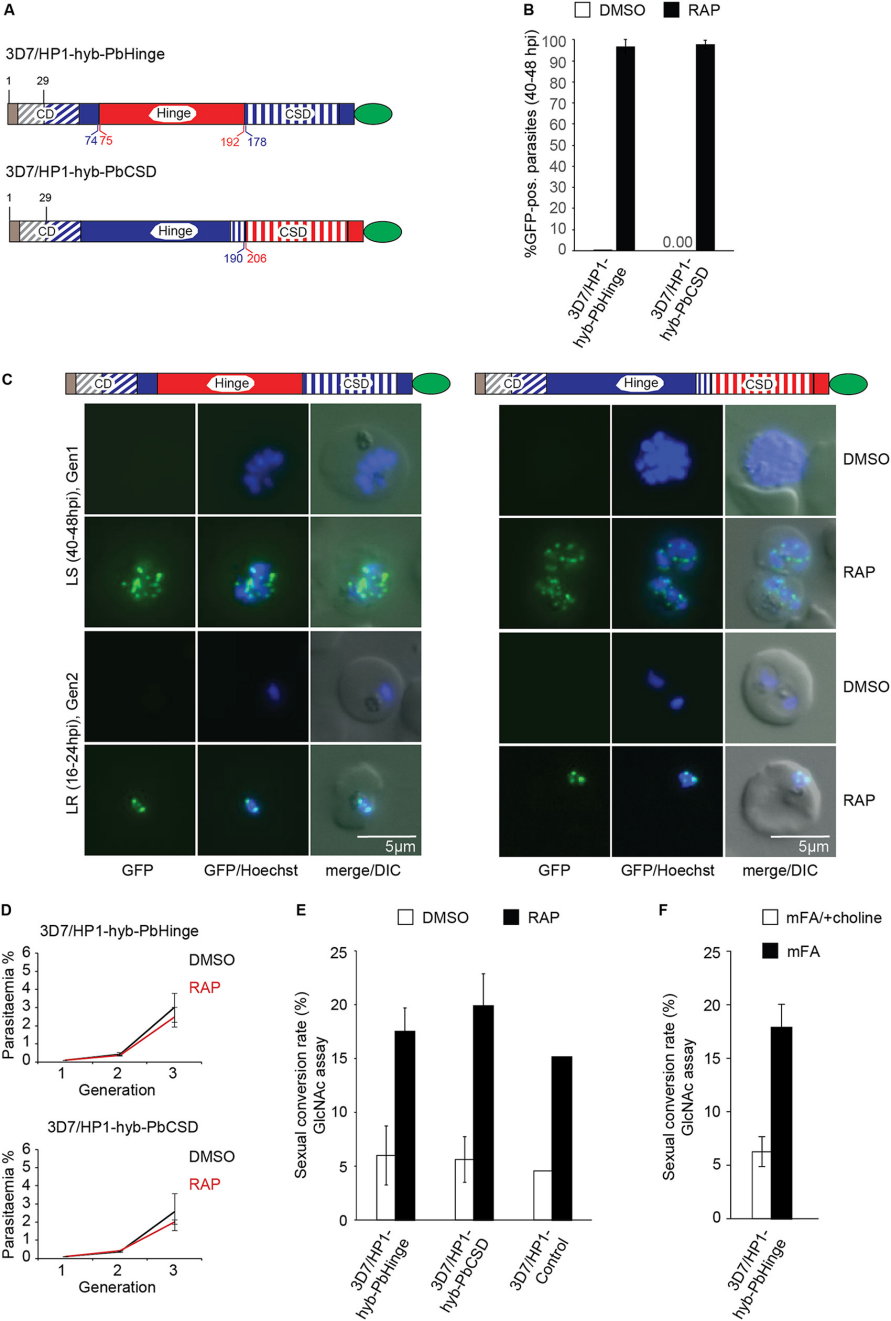
Generation of DiCre-inducible PfHP1-PbHP1 hybrid mutants. (A) Diagrams showing the GFP-tagged PfHP1-PbHP1 hybrid proteins expressed in the 3D7/HP1-hyb-PbHinge and 3D7/HP1-hyb-PbCSD cell lines after RAP treatment. Brown and blue colors represent the remaining wild-type PfHP1 N terminus and the replacing PfHP1 protein sequences, respectively. Red colors identify the hinge domain and CSD derived from PbHP1. The CD and CSD are indicated by diagonal and vertical dashed stripes, respectively. Numbers in blue and red refer to amino acid positions within the PfHP1 and PbHP1 sequences, respectively. (B) Proportions of GFP-positive parasites observed 40 h after treatment with RAP or DMSO (control). Values represent the means from three independent biological replicates (error bars indicate SD). For each sample, >140 iRBCs were counted. (C) Representative live-cell fluorescence images showing the localization of GFP-tagged PfHP1-PbHP1 hybrid proteins in 3D7/HP1-hyb-PbHinge and 3D7/HP1-hyb-PbCSD parasites in late schizonts (LS) (40 to 48 hpi; generation 1 [Gen1], 40 h after RAP treatment) and in late-ring-stage progeny (LR) (16 to 24 hpi, generation 2). Nuclei were stained with Hoechst dye. DIC, differential interference contrast. Scale bar, 5 μm. (D) Growth curves of the DMSO- and RAP-treated 3D7/HP1-hyb-PbHinge and 3D7/HP1-hyb-PbCSD parasites over three consecutive generations. Values are the means from at least three independent replicate experiments (error bars represent SD). For each sample, >3,000 RBCs were counted. (E) Sexual conversion rates of the DMSO- and RAP-treated 3D7/HP1-hyb-PbHinge and 3D7/HP1-hyb-PbCSD mutants and the 3D7/HP1-Control line, assessed by inspection of Giemsa-stained blood smears of GlcNAc-treated cultures on day 6 of gametocytogenesis. Values represent the means from at least three independent replicate experiments (error bars represent SD). The values for the 3D7/HP1-Control line derive from a single experiment and are consistent with previously published data (69). For each sample, >3,000 RBCs were counted. (F) Sexual conversion rates of 3D7/HP1-hyb-PbHinge parasites cultured in minimal fatty acid medium (mFA) or mFA supplemented with 2 mM choline (mFA/+choline), assessed by inspection of Giemsa-stained blood smears of GlcNAc-treated cultures on day 6 of gametocytogenesis. Values represent the means from two independent replicate experiments (error bars represent SD). For each sample, >1,800 RBCs were counted.

PfHP1 (266 amino acids) and PbHP1 (281 amino acids) display an overall sequence identity of 68%. The CDs and CSDs are highly conserved, with 88% and 90% identical amino acids, respectively, whereas the intervening sequence encompassing the hinge domain shows poor conservation, with only 45% sequence identity (Fig. S2). We first attempted to obtain a transgenic line where RAP treatment would replace *pfhp1* with the *pbhp1* gene. For unknown reasons, however, we repeatedly failed to integrate the corresponding conditional expression cassette into the endogenous *pfhp1* locus. Hence, we decided to perform domain swap experiments and generated two DiCre-inducible PfHP1 hybrid cell lines, namely, 3D7/HP1-hyb-PbHinge and 3D7/HP1-hyb-PbCSD. In these cell lines, RAP treatment leads to the replacement of the PfHP1 hinge domain or CSD with the PbHP1 hinge domain or CSD, respectively, and both hybrid HP1 proteins are expressed as C-terminal GFP fusions (Fig. 4A and Fig. S1). Note that a CD swap cell line could not be generated for technical reasons, as the 3D7/N31DC mother line used to generate the conditional mutants does not allow one to swap the entire CD (Fig. S1). PCR of gDNA confirmed the correct insertion of the two hybrid *hp1* sequences downstream of the endogenous *pfhp1* gene and the successful replacement of endogenous *pfhp1* with the hybrid genes after RAP-induced DiCre-mediated recombination in both lines (Fig. S1). To assess the ability of the HP1 hybrid proteins in forming heterochromatic domains, we performed live-cell fluorescence imaging at 40 to 48 hpi (generation 1, 40 h after RAP treatment) and at 16 to 24 hpi in generation 2 ring stages to observe the localization of the GFP fusion proteins. As expected, no GFP signal was detectable in either DMSO-treated control parasite (Fig. 4B and C). After RAP treatment, over 95% of parasites expressed the GFP-tagged hybrid HP1 proteins (Fig. 4B), and both PfHP1-hyb-PbHinge-GFP and PfHP1-hyb-PbCSD-GFP localized to perinuclear heterochromatin foci in schizonts and in the ring-stage progeny in a pattern indistinguishable from that observed for wild-type PfHP1-GFP (49, 60, 69) (Fig. 2 and 4C). Thus, replacement of the PfHP1 hinge domain or CSD with those from PbHP1 retains proper heterochromatin localization of HP1 in P. falciparum.

10.1128/mSphere.01220-20.3 FIG S2 Pairwise sequence alignment of PfHP1 and PbHP1. Alignment of the PfHP1 and PbHP1 amino acid sequences by EMBOSS Needle (https://www.ebi.ac.uk/Tools/psa/emboss_needle/). Identical amino acids are marked by asterisks. The PfHP1 CD and CSD are highlighted by horizontal lines; purple hashtags denote residues important for H3K9me binding by the CD, and blue and green hashtags denote residues important for CSD homo- and heterodimerization, respectively (12). Download FIG S2, TIF file, 0.7 MB. Copyright © 2021 Bui et al. 2021 Bui et al. This content is distributed under the terms of the Creative Commons Attribution 4.0 International license.

We next evaluated parasite proliferation in the 3D7/HP1-hyb-PbHinge and 3D7/HP1-hyb-PbCSD lines over three generations and observed no major differences in parasite multiplication between DMSO- and RAP-treated parasites, revealing the proper function of the HP1 hybrid proteins in controlling mitotic cell cycle progression and parasite proliferation (Fig. 4D). This observation also indicated that the HP1 hybrid proteins mediate efficient silencing of the *pfap2-g* locus. To confirm this property, we compared the SCRs of DMSO- and RAP-treated parasites for both HP1 hybrid lines and the 3D7/HP1-Control line using the GlcNAc assays as described above. As shown in Fig. 4E, the RAP-treated 3D7/HP1-hyb-PbHinge and 3D7/HP1-hyb-PbCSD populations showed a 3- to 4-fold-increased SCR compared to that of the DMSO-treated controls. However, RAP-treated 3D7/HP1-Control parasites also showed a 3- to 4-fold-increased SCR in the RAP treatment cycle, which has been observed previously and is caused by unknown mechanisms linked to the DiCre-mediated exchange of endogenous *pfhp1* with the recodonized *pfhp1-gfp* gene (69). Hence, the PfHP1-hyb-PbHinge and PfHP1-hyb-PbCSD proteins appear to silence the *pfap2-g* locus as efficiently as wild-type PfHP1. Furthermore, since 3D7/HP1-hyb-PbHinge and 3D7/HP1-hyb-PbCSD parasites were still able to produce gametocytes at a rate similar to that of the 3D7/HP1-Control line, it seems that GDV1 can interact with and evict the hybrid HP1 proteins from the *pfap2-g* locus. To confirm this hypothesis, we induced sexual commitment in 3D7/HP1-hyb-PbHinge parasites using minimal fatty acid (mFA) medium lacking lysophosphatidylcholine (lysoPC) and choline (66). While the exact mechanisms involved in this sensing pathway remain elusive, the increased sexual commitment rates observed under lysoPC/choline-depleted conditions correlates with an increased proportion of parasites expressing GDV1 (60). To this end, 3D7/HP1-hyb-PbHinge parasites were treated with RAP to swap endogenous *pfhp1* with the *pfhp1-hyb-pbhinge* hybrid gene. After one additional multiplication cycle, PfHP1-hyb-PbHinge-expressing parasites were split and cultured separately in mFA (induces sexual commitment) or mFA supplemented with 2 mM choline (mFA/+choline) (suppresses sexual commitment) (66), and SCRs were determined in the progeny using the GlcNAc assay. As shown in Fig. 4F, 3D7/HP1-hyb-PbHinge parasites were indeed responsive to choline depletion, showing a 3-fold-increased SCR compared to that of the same population grown in the presence of choline.

In summary, even though we failed to generate a P. falciparum line expressing full-length PbHP1, our findings obtained with the hinge domain and CSD swap mutants demonstrate that PbHP1 can execute the core functions of PfHP1 in proliferation and gene silencing in P. falciparum parasites. They, furthermore, suggest that PbHP1 can still functionally interact with GDV1 even though P. berghei parasites do not possess a GDV1 ortholog.

## DISCUSSION

Here, we used a DiCre-based conditional protein domain deletion and swapping approach to study the function of PfHP1 in P. falciparum blood-stage parasites. By combining the data gained from subcellular protein localization analysis and parasite multiplication and sexual differentiation assays, we are able to draw important conclusions as to the specific roles that each domain plays in mediating proper PfHP1 function.

Previous studies indicated that in different eukaryotes, different domains of HP1 carry the sequence information required for targeting HP1 to the nucleus and that these regions do not always possess canonical NLS sequences (19, 26–28, 75, 76). Therefore, we could not rely on sequence homology to predict the nucleus-targeting sequences within PfHP1 but had to determine them experimentally instead. We found that the 76 C-terminal residues encoding the CSD (amino acids 191 to 266) are essential for effective nuclear targeting, as only the PfHP1-ΔCD and PfHP1-ΔHinge fusions, not the PfHP1-ΔCSD fusion, were efficiently imported into the nucleus. It is possible that the predicted NLS motif in the CSD (RRKK; amino acids 201 to 204) is indeed directly responsible for directing PfHP1 into the nucleus. The predicted NLS element in the hinge domain (PRRK; amino acids 100 to 103) may also allow some level of nuclear import, as a fraction of PfHP1-ΔCSD proteins still localized to the nucleus. However, since the bioinformatic identification of NLSs in P. falciparum proteins has only weak predictive power (77), mutational analyses of these putative NLS motifs will be required to test whether and to what extent they are implicated in mediating the nuclear import of PfHP1.

Interestingly, none of the three PfHP1 domain deletion mutants was capable of forming and inheriting heterochromatin to daughter nuclei during schizogony. This conclusion is based on the observation that neither the PfHP1-ΔCD and PfHP1-ΔHinge proteins, both of which localized exclusively to the nucleus, nor the nucleus-localized pool of the PfHP1-ΔCSD mutant formed perinuclear heterochromatic foci. Heterochromatin binding of HP1 has been well investigated in many model organisms (19, 26–28, 76). Studies employing different experimental systems highlighted the contribution of more than one structural HP1 domain for proper heterochromatin targeting. In mice, for instance, the heterochromatin-targeting ability of HP1α involves RNA binding via a region in the hinge domain together with binding to methylated histone 3 via the CD (19). In D. melanogaster, the domains targeting HP1 to the nucleus and to heterochromatin were identified by analyzing a panel of HP1 truncation mutants tagged with β-galactosidase at the N terminus (27). As in our study, protein fusions containing the CD and/or hinge domain but lacking the CSD failed to localize to the nucleus, while protein fusions containing the majority of the CSD region (amino acids 152 to 206 [the CSD encompasses amino acids 142 to 206]) showed nuclear localization. However, proper assembly into heterochromatin was found only for protein fusions containing a substantial part of the hinge domain and the CSD region (amino acids 95 to 206) (27). Given that in these experiments the β-galactosidase–HP1 fusions were ectopically expressed in a wild-type HP1 background, successful heterochromatin targeting may have resulted from hetero-dimerization between CSD-containing fusion proteins and endogenous HP1. In our study, however, the PfHP1-GFP truncation mutants were expressed in a PfHP1 null background and only full-length PfHP1 was able to assemble into perinuclear heterochromatic foci during schizogony. To interpret these findings, it is useful to bring to mind the process of parasite multiplication by schizogony, where four to five subsequent rounds of genome duplication and nuclear division precede daughter cell formation. The reestablishment of heterochromatin on newly replicated chromosomes is likely initiated by the binding of PfHP1 to existing H3K9me3-containing nucleosomes that are distributed between the replicated chromosomes in a semiconservative manner. Heterochromatin spreading, however, will require *de novo* methylation of H3K9 on newly incorporated nucleosomes by a H3K9-specific SUVAR3-9-like HKMT (presumably PfSET3 [36, 78, 79]), the recruitment of which depends on its interaction with the CSD of HP1 (5, 17). Hence, mutations that prevent PfHP1 either from binding to chromatin or from recruiting the H3K9-specific HKMT will equally result in defective heterochromatin formation, and this defect will become more pronounced with each additional round of genome duplication during schizogony. The fact that the PfHP1ΔCD-GFP, PfHP1ΔHinge-GFP, and PfHP1ΔCSD-GFP deletion mutants all failed to assemble into perinuclear heterochromatic foci in schizonts and the subsequent ring-stage progeny shows that each of the three domains is essential for the *de novo* heterochromatin assembly on replicated chromosomes. While this observation is not surprising with regard to the CD (required for binding to H3K9me3) and CSD (required for PfHP1 dimerization and HKMT recruitment), our results demonstrate that the PfHP1 hinge domain is also indispensable for proper heterochromatin assembly. Studies on mouse and Xenopus laevis HP1 proteins have shown that the RNA- and DNA-binding capacity of the hinge domain is important for high-affinity binding of HP1 to H3K9me3 and stable heterochromatin formation (18–20). We therefore anticipate that the crucial role of the PfHP1 hinge domain in heterochromatin assembly may be based on similar properties.

Conditional expression of the three PfHP1 domain deletion mutants phenocopied the conditional knockout of PfHP1 in the 3D7/HP1-KO line. All four mutants produced heterochromatin-depleted ring-stage progeny that consisted of up to 95% sexually committed parasites and a small proportion of asexual parasites that failed to proliferate further. This phenotype is identical to but more pronounced than that obtained upon knocking down PfHP1-GFP-DD expression (49). In their previous study, Brancucci and colleagues demonstrated that conditional depletion of PfHP1 expression released the *pfap2-g* locus from PfHP1-dependent silencing, which in turn triggered PfAP2-G expression and sexual conversion in 52% of the ring-stage progeny. The other half of the progeny represented asexual parasites that arrested at the trophozoite stage due to failed entry into S phase (49). The substantially higher proportion of sexually committed ring-stage progeny observed in the inducible PfHP1 knockout (KO) or domain deletion mutants generated here shows that the DiCre-mediated excision or mutagenesis of the *pfhp1* gene leads to an almost complete abolishment of PfHP1-dependent gene silencing. Furthermore, these results also demonstrate that the lack of perinuclear heterochromatic foci in the PfHP1 domain deletion mutants is also reflected at the functional level in defective gene silencing.

Previous chromatin immunoprecipitation followed by next-generation sequencing (ChIP-seq) experiments performed on six different *Plasmodium* species (P. falciparum, P. vivax, P. knowlesi, P. berghei, P. yoelii, P. chabaudi) suggest a conserved role for heterochromatin in facilitating the clonally variant expression of species-, clade-, and genus-specific genes that are involved primarily in host-parasite interactions during blood-stage infection (29, 30). In addition, HP1 also controls the heritable silencing of *ap2-g* in all malaria parasite species examined to date (29, 30). In P. falciparum, activation of *pfap2-g* expression and subsequent sexual conversion is triggered by the GDV1-dependent displacement of PfHP1 from the *pfap2-g* locus (60). Notably, GDV1 is essential for this process because GDV1 loss-of-function mutants are unable to commit to gametocytogenesis (62, 65, 80). Expression of GDV1 itself appears to be negatively regulated by a long noncoding antisense RNA (60, 81), and low concentrations of the serum lipid lysoPC induce GDV1 expression through an unknown sensing pathway (60). In addition to P. falciparum, all other *Plasmodium* spp. infecting primates as well as avian malaria parasites (Plasmodium gallinaceum, Plasmodium relictum) possess a GDV1 ortholog (https://plasmodb.org/plasmo/app), suggesting that these species may employ a conserved strategy to activate *ap2-g* expression in response to environmental stimuli. Intriguingly, however, *Plasmodium* spp. infecting rodents lost the *gdv1* locus (60, 65, 66), and sexual commitment in P. berghei is insensitive to lysoPC depletion (66). Whether *ap2-g* activation in these parasites requires an unknown factor functionally equivalent to GDV1 and responsive to an alternative sensing pathway, or whether PbHP1 is less stably associated with the *pbap2-g* locus, allowing for a higher level of stochastic activation, is unknown at this stage. Our results show that the hinge domain and the CSD of PbHP1 can fully complement PfHP1’s function in mitotic proliferation, heterochromatin formation, and *pfap2-g* silencing. Because 3D7/HP1-hyb-PbHinge and 3D7/HP1-hyb-PbCSD parasites did not display higher default SCRs than the 3D7/HP1-Control line, both hybrid HP1 proteins seem to form stable heterochromatin domains, similarly to wild-type PfHP1, at least in P. falciparum. Importantly, our findings also demonstrate that GDV1 can still recognize and displace both HP1 hybrid proteins from the *pfap2-g* locus. Although the physical interaction between GDV1 and PfHP1 has not yet been mapped to a particular PfHP1 domain (60), we speculate that GDV1 may interact with the CSD for two reasons. First, the CSD dimer interface is responsible for most interactions between HP1 and other regulatory factors in model eukaryotes (7–9, 11). Second, in contrast to their hinge domains, the CSDs of PfHP1 and PbHP1 share high sequence identity and perfect conservation of residues predicted to be involved in CSD homo-dimerization and protein-protein interactions (Fig. S2). However, we cannot rule out the possibility that the N terminus and/or the CD is important for this interaction. Irrespective of this uncertainty, our findings strongly suggest that PbHP1 retained the capacity to interact with GDV1 and that this interaction evolved based on evolutionarily conserved features of HP1 orthologs in malaria parasites. In light of this conclusion, it may be interesting to explore whether ectopic expression of GDV1 can be used as an experimental tool for the induction of high sexual conversion rates in P. berghei, as was recently reported for P. falciparum (58, 60).

In summary, we demonstrate that each of the three PfHP1 domains is essential for heterochromatin formation, gene silencing, and mitotic proliferation in P. falciparum blood-stage parasites. We also discovered that the hinge domain and CSD of P. berghei HP1 fully complement the function of the corresponding domains of PfHP1 in these processes and sustain the capacity for GDV1-dependent eviction of HP1 from the *pfap2-g* locus. Together, these findings provide major new insight into HP1 function in malaria parasites and offer new possibilities for a further functional dissection of this essential silencing factor in parasite biology.

## MATERIALS AND METHODS

### Parasite culture and transfection.

The transgenic cell lines generated in this study were cultured at 5% hematocrit in RPMI 1640 medium supplemented with 25 mM HEPES, 0.45 mM hypoxanthine, 24 mM sodium bicarbonate, and 0.5% AlbuMAX II supplemented with 2 mM choline to reduce default sexual conversion rates (SCRs) as demonstrated recently (66). Parasite cultures were synchronized using 5% sorbitol as described previously (82). Cotransfection of CRISPR/Cas9 and donor plasmids into the DiCre-expressing line 3D7/1G5DiCre (68) and selection of transfected populations were performed as described recently (60, 69).

### Transfection constructs.

We applied CRISPR/Cas9-mediated genome editing and the DiCre/loxP system (67, 68) to generate parasite lines conditionally expressing truncated PfHP1 and hybrid HP1 variants. We engineered (i) 3D7/HP1-KO for full-length PfHP1 (PF3D7_1220900) deletion (deletion of amino acids 30 to 266); (ii) 3D7/HP1-ΔCD for expression of a PfHP1 CD deletion mutant (deletion of amino acids 30 to 58); (iii) 3D7/HP1-ΔHinge for expression of a PfHP1 hinge domain deletion mutant (amino acids 75 to 177 of PfHP1 were replaced with a linker peptide representing amino acids 232 to 254 of PfSIP2 [PF3D7_0604100]); (iv) 3D7/HP1-ΔCSD for expression of a PfHP1 CSD deletion mutant (deletion of amino acids 191 to 266); (v) 3D7/HP1-hyb-PbHinge for expression of a chimeric PfHP1, in which the PfHP1 hinge domain (amino acids 75 to 177) was replaced by the PbHP1 (PBANKA_1436100) hinge domain (amino acids 75 to 192); and (vi) 3D7/HP1-hyb-PbCSD for expression of a chimeric PfHP1, in which the PfHP1 CSD (amino acids 191 to 266) was replaced by the PbHP1 CSD (amino acids 206 to 281). To obtain these cell lines, we transfected the mother cell line 3D7-1G5DC/5′-loxPint-g31 (or 3D7/N31DC), which carries a *sera2* intron:loxP element (67) within the 5′ end of the *pfhp1* coding sequence (69), with donor plasmids to insert a second *sera2* intron:loxP sequence directly downstream of the endogenous *pfhp1* stop codon, followed by either the *gfp* coding sequence (HP1-KO) or a recodonized mutated *pfhp1* sequence fused to *gfp* (PfHP1-ΔCD, PfHP1-ΔHinge, PfHP1-ΔCSD, PfHP1-hyb-PbHinge, PfHP1-hyb-PbCSD). The donor plasmids are derivatives of the pD-PfHP1-Control donor plasmid described recently (69). Each donor plasmid was cotransfected with the CRISPR/Cas9 plasmid pBF-gC-guide250, which expresses SpCas9, a guide RNA targeting the 3′ end of the *pfhp1* coding sequence and the positive-negative drug selection marker blasticidin deaminase (BSD) fused to yeast cytosine deaminase/uridyl phosphoribosyl transferase (yFCU) (69).

Cloning of the pD-PfHP1-KO plasmid has recently been described (69) and consists of the pUC19 backbone carrying a 5′ homology region (HR) spanning bp +88 to +798 of the *pfhp1* gene, terminating with a stop codon, followed by the 103-bp sera2 intron:loxP element (67), the *gfp* coding sequence ending with a stop codon, and a 3′ HR encompassing the 824 bp directly downstream of the *pfhp1* coding sequence. All additional donor plasmids described below carry the same 5′ and 3′ HR for homology-directed repair of the Cas9-induced DNA lesion.

The pD-PfHP1-ΔCD donor plasmid was constructed by Gibson assembly joining of three PCR fragments encoding (i) the pD plasmid backbone amplified from pUC19 using primers PCRA_F and PCRA_R, (ii) the *pfhp1* 5′ HR followed by the 103-bp *sera2* intron:loxP sequence amplified from pD-PfHP1-Control (69) using primers F158 and R143, and (iii) a fragment amplified from pD-PfHP1-Control using primers F177 and R163 spanning, in the following order, bp +175 to +798 of a synthetic recodonized *pfhp1* coding sequence (69) omitting the stop codon, the *gfp* coding sequence ending with a stop codon, and the *pfhp1* 3′ HR.

The pD-PfHP1-ΔHinge donor plasmid was constructed by Gibson assembly joining four PCR fragments encoding (i) the pD plasmid backbone amplified from pUC19 using primers PCRA_F and PCRA_R; (ii) the *pfhp1* 5′ HR followed by the *sera2* intron:loxP sequence amplified from pD-PfHP1-Control using primers F158 and R143; (iii) a fragment amplified from the pBcam-ΔHinge-3HA-Cherry plasmid (see Text S1 in the supplemental material) using primers F164 and R165 spanning, in the following order, bp +88 to +222 of the recodonized *pfhp1* sequence, bp +694 to +762 of the *pfsip2* coding sequence encoding the linker region separating the tandem AP2 domains (70), and bp +532 to +798 of the recodonized *pfhp1* coding sequence omitting the stop codon; and (iv) a fragment amplified from pFdon-C-loxP-g250 (69) using primers F162 and R163, spanning the *gfp* coding sequence and *pfhp1* 3′ HR.

10.1128/mSphere.01220-20.1 TEXT S1 Supplementary methods. Download Text S1, PDF file, 0.1 MB. Copyright © 2021 Bui et al. 2021 Bui et al. This content is distributed under the terms of the Creative Commons Attribution 4.0 International license.

The pD-PfHP1-ΔCSD donor plasmid was constructed by Gibson assembly joining three PCR fragments encoding (i) the pD plasmid backbone amplified from pUC19 using primers PCRA_F and PCRA_R; (ii) a fragment amplified from pD-PfHP1-Control using primers F158 and R178 spanning, in the following order, the *pfhp1* 5′ HR followed by the *sera2* intron:loxP element and bp +88 to +570 of the recodonized *pfhp1* coding sequence; and (iii) a fragment amplified from pFdon-C-loxP-g250 (69) using primers F162 and R163, spanning the *gfp* coding sequence and *pfhp1* 3′HR.

The pD-hyb-PbHinge donor plasmid was constructed by Gibson assembly joining two PCR fragments encoding (i) part of the pD-HP1-KO plasmid amplified from its own template using primers F162 and R143 and representing, in the following order, the *gfp* coding sequence, the *pfhp1* 3′ HR, the pUC19 plasmid backbone, and the pfhp1 5′ HR followed by the 103-bp sera2 intron:loxP sequence; and (ii) a fragment amplified from the pBcam-hyb-PbHinge-3HA-Cherry plasmid (Text S1) using primers F164 and R165 spanning, in the following order, bp +88 to +222 of the recodonized *pfhp1* coding sequence, bp +223 to +576 of the *pbhp1* coding sequence, and bp +532 to +798 of the recodonized *pfhp1* coding sequence, omitting the stop codon.

The pD-hyb-PbCSD donor plasmid was constructed by Gibson assembly as explained above for pD-hyb-PbHinge joining two PCR fragments encoding (i) the corresponding part of the pD-HP1-KO plasmid backbone amplified from its own template using primers F162 and R143 and (ii) a fragment amplified from pBcam-hyb-PbCSD-3HA-Cherry (Text S1) using primers F164 and R161, spanning bp +88 to +570 of the recodonized *pfhp1* coding sequence followed by bp +616 to +843 of the *pbhp1* sequence, omitting the stop codon.

For each transfection, 50 μg of the pBF-gC-guide250 plasmid was mixed with 50 μg of donor plasmid and electroporated into the 3D7/N31DC mother parasite line. Transfected parasites were selected on 5 μg/ml BSD-S-HCl and established as described previously (60). All oligonucleotide sequences used for the cloning of donor plasmids are provided in Table S1.

10.1128/mSphere.01220-20.4 TABLE S1 Oligonucleotides used in this study. Download Table S1, PDF file, 0.09 MB. Copyright © 2021 Bui et al. 2021 Bui et al. This content is distributed under the terms of the Creative Commons Attribution 4.0 International license.

### Induction of DiCre recombinase-mediated DNA excision by rapamycin treatment.

Parasites were synchronized twice 16 h apart to obtain an 8-h growth window (16 to 24 hpi). After invasion into new RBCs, parasites were synchronized again at 0 to 8 hpi (generation 1) and split into two equal populations, one of which was treated with 0.02% DMSO (negative control) and the other of which was treated with 100 nM RAP for 60 to 90 min as described previously (83). The cultures were then spun down, washed once, and resuspended in culture medium lacking RAP for onward *in vitro* culture.

### Live-cell fluorescence imaging and indirect immunofluorescence assays.

To quantify the efficiency of *pfhp1* excision after RAP treatment, live-cell fluorescence microscopy was performed as described before (84) with a minor modification using 5 μg/ml Hoechst dye (Merck) to stain the nuclei. Excision efficiency was determined as the percentage of GFP-positive schizonts at 40 to 48 hpi in generation 1 (40 h after RAP treatment) (>200 schizonts were counted per experiment). IFAs were performed on methanol-fixed cells using mouse monoclonal antibody (MAb) anti-Pfs16 (kind gift from Robert W. Sauerwein) at 1:250 and Alexa Fluor 488-conjugated anti-mouse IgG (Molecular Probes) at 1:250. Nuclei were stained with 5 μg/ml Hoechst dye (Merck). Images were taken at a 630-fold magnification on a Leica DM 5000B microscope with a Leica DFC 300 FX camera, acquired via the Leica IM1000 software and processed using ImageJ (https://imagej.nih.gov/ij/). For each experiment, images were acquired and processed with identical settings.

### Parasite multiplication assay.

Parasites were tightly synchronized twice 16 h apart within the same intraerythrocytic cycle. After invasion into new RBCs, parasites were split at 0 to 8 hpi (generation 1) into two equal populations, one half of which was treated with 0.02% DMSO (negative control) and the other half of which was induced for DiCre recombinase-mediated DNA excision by RAP treatment as described above. Giemsa smears were prepared to determine the parasitemia at 16 to 24 hpi (generation 1). Giemsa-stained smears were also prepared 2 days later (generation 2) and 4 days later (generation 3). Parasitemia was counted by visual inspection of Giemsa-stained blood smears (≥3,000 RBCs counted per experiment). Parasite multiplication rates (PMRs) were determined as the parasitemia observed in the following generation divided by the parasitemia observed in the previous generation.

### Gametocyte conversion assay.

After DMSO or RAP treatment in generation 1, parasites were allowed to complete schizogony and reinvade RBCs. At 16 to 24 hpi in generation 2 (day 1 of gametocytogenesis), each pair of parasites was treated with 50 mM *N-*acetyl-d-glucosamine (GlcNAc) for 6 days to eliminate asexual parasites (72, 73) and then cultured with normal culture medium for another 6 days to observe the maturation of gametocytes. Gametocytemia was determined on day 6 of GlcNAc treatment by visual inspection of Giemsa-stained blood smears. SCRs were determined as the gametocytemia observed on day 6 of gametocyte development as a proportion of the total parasitemia observed on day 1 of gametocytogenesis. For the experiment presented in Fig. 4F, 3D7/HP1-hyb-PbHinge ring-stage parasites (generation 1) were treated with RAP to swap endogenous *pfhp1* with the *pfhp1-hyb-pbhinge* hybrid gene and allowed to replicate and invade new RBCs. At 24 to 30 hpi (generation 2), the population was split and cultured separately in minimal fatty acid medium (mFA) or mFA supplemented with 2 mM choline (mFA/+choline) to induce or suppress sexual commitment, respectively, as previously described (60, 66). At 16 to 24 hpi in generation 3 (day 1 of gametocytogenesis), the paired populations were cultured in the presence of 50 mM GlcNAc until day 6 of gametocyte development and SCRs were determined as described above.

### Genomic DNA isolation, PCR, and Sanger sequencing.

To evaluate correct editing of the *pfhp1* locus, PCRs of gDNA isolated from all transgenic parasite lines were performed. gDNAs were sampled and isolated as described previously (84). To evaluate the DNA excision efficiency after RAP treatment, PCRs were performed on gDNA isolated 24 to 36 h posttreatment. Primers were designed to allow PCR amplification across the 5′-to-3′ homology regions. All transfection plasmids generated in this study have been validated by Sanger sequencing. All transfection plasmids have been designed and Sanger sequencing results analyzed using the SnapGene software (GSL Biotech). All PCR primer sequences are listed in Table S1.
