# Supplementary figures and images for: Investigation of Heterochromatin Protein 1 Function in the Malaria Parasite Plasmodium falciparum Using a Conditional Domain Deletion and Swapping Approach
Source: mSphere. 2021 Feb 3;6(1):e01220-20. doi: 10.1128/mSphere.01220-20 (PMC7860992; doi:10.1128/mSphere.01220-20)

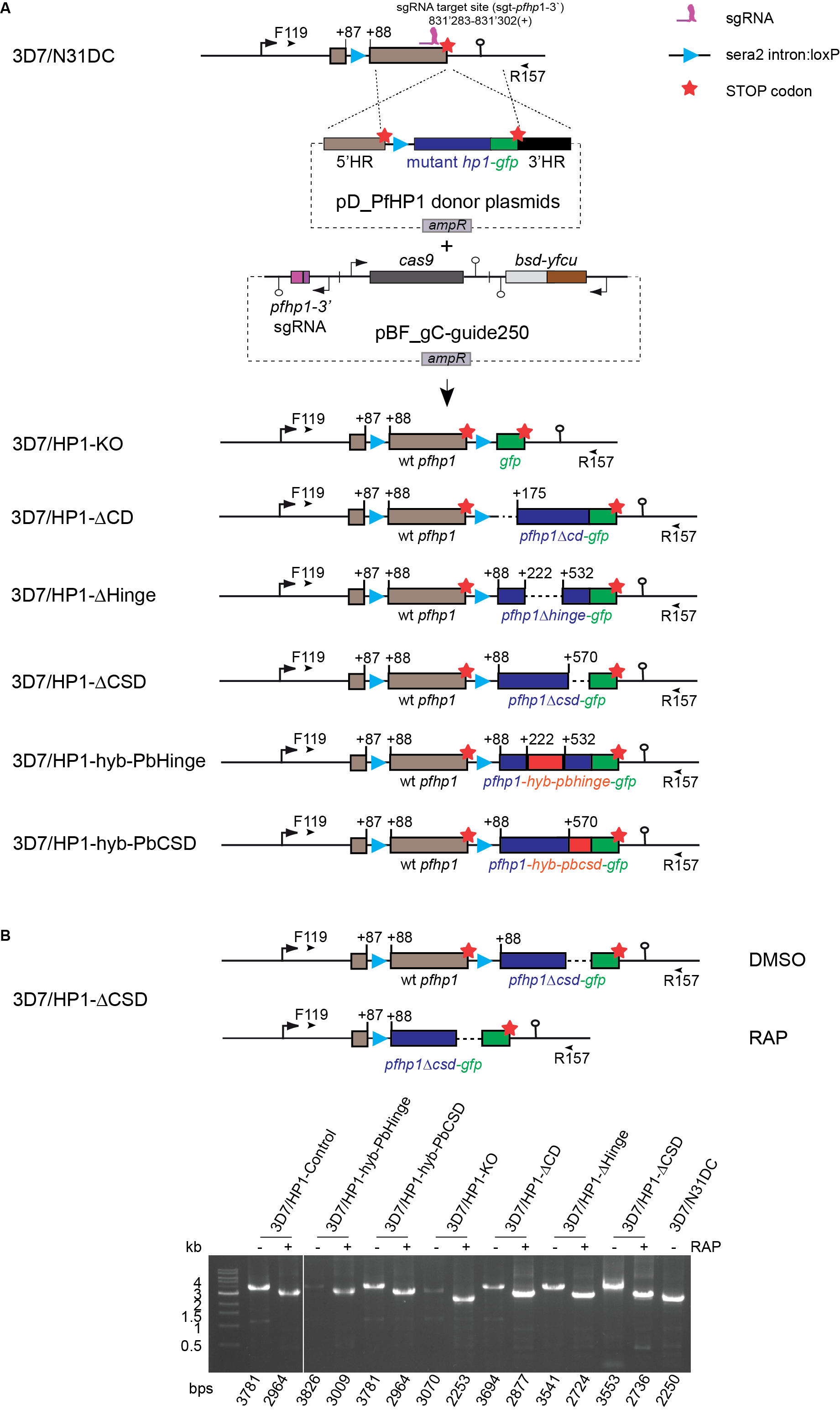

Supplement: FIG S1 [file mSphere.01220-20-sf001.tif]

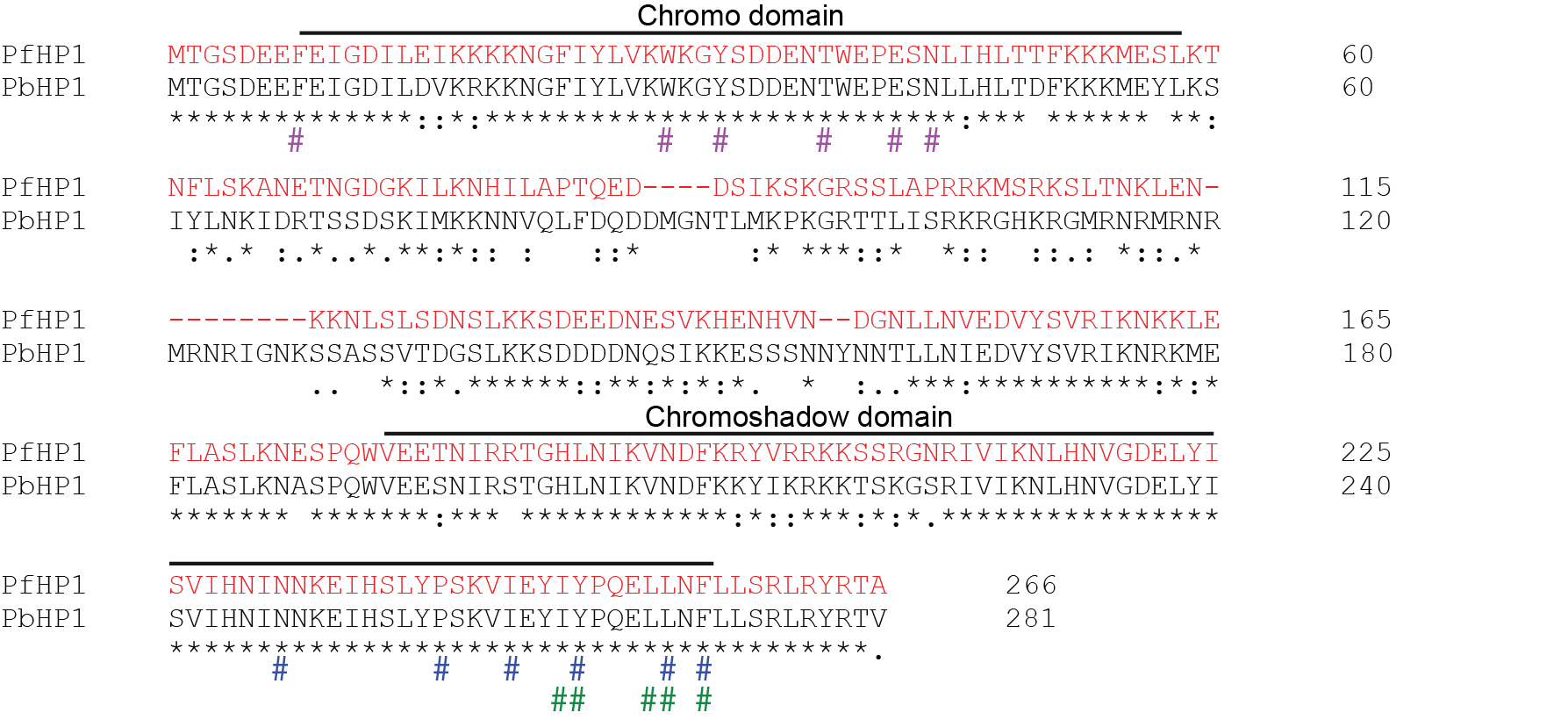

Supplement: FIG S2 [file mSphere.01220-20-sf002.tif]
