# Supplementary material for: Investigation of Heterochromatin Protein 1 Function in the Malaria Parasite Plasmodium falciparum Using a Conditional Domain Deletion and Swapping Approach
Source: mSphere. 2021 Feb 3;6(1):e01220-20. doi: 10.1128/mSphere.01220-20 (PMC7860992; doi:10.1128/mSphere.01220-20)
Supplement: TEXT S1 [file mSphere.01220-20-s0001.pdf]

TEXT S1. Supplementary Methods

Generation of the pBcam-ΔHinge-3HA-Cherry, pBcam-hyb-PbHinge-3HA-Cherry and pBcam-hyb-PbCSD-3HA-Cherry plasmids. The pBcam-ΔHinge-3HA-Cherry plasmid was constructed as follows. The fragment PfCD spanning bps +1 to +222 of the recodonised *pfhp1* sequence was amplified from a pUC57 plasmid containing a synthetic recodonized *pfhp1* coding sequence (pUC57-re-*pfhp1)* (70) using primers F11 and R148. The fragment PfSIP2.linker (spanning bps +694 to +762 of the *pfsip2* coding sequence) was amplified from 3D7 gDNA using primers F4 and R5. The fragment PfCSD spanning bps +532 to +798 of the recodonized *pfhp1* sequence and omitting the stop codon was amplified from pUC57-re-*pfhp1* using primers F2 and R3. A hybrid fragment consisting of the PfSIP2.linker and PfCSD fragments was amplified by fusion PCR from a mixture of the PfSIP2.linker and PfCSD PCR templates using primers F4 and R3. The final hybrid fragment of PfCD/PfSIP2.linker/PfCSD was amplified by fusion PCR from a mixture of PfCD and PfSIP2.linker/PfCSD PCR templates using primers F11 and R3 and cloned into pBcam-3HA-Cherry (85) after digestion with *BamH*I and *Nhe*I and ligation with T4 DNA ligase.

The pBcam-hyb-PbHinge-3HA-Cherry plasmid was constructed as follows. The fragment PfCD spanning bps +1 to +222 of the recodonized *pfhp1* sequence was amplified from pUC57-re-*pfhp1* using primers F11 and R1. The fragment PbHinge spanning bps +223 to +576 of the *pbhp1* sequence was amplified from *P. berghei* gDNA using primers F35 and R36. The fragment PfCSD spanning bps +532 to +798 of the recodonized *pfhp1* sequence and omitting stop codon was amplified from pUC57-re-*pfhp1* using primers F2 and R42. A hybrid fragment consisting of the PfCD/PbHinge fragments was amplified by fusion PCR from a mixture of PfCD and PbHinge PCR templates using primers F11 and R36. The final hybrid fragment of PfCD/PbHinge/PfCSD was amplified by fusion PCR from a mixture of the PfCD/PbHinge and PfCSD PCR templates using primers F11 and R42 and cloned into pBcam-3HA-Cherry after digestion with *BamH*I and *Not*I and ligation with T4 DNA ligase.

The pBcam-hyb-PbCSD-3HA-Cherry plasmid was constructed as follows. The fragment PfCD.Hinge spanning bps +1 to +570 of the recodonized *pfhp1* sequence was amplified from pUC57-re-*pfhp1* using primers F11 and R12. The fragment PbCSD spanning bps +616 to +843 of the *pbhp1* sequence and omitting stop codon was amplified from *P. berghei* gDNA using primers F38 and R41. A hybrid fragment consisting of PfCD.Hinge/PbCSD was amplified by fusion PCR from a mixture of the PfCD.Hinge and PbCSD PCR templates using primers F11 and R41 and cloned into pBcam-3HA-Cherry after digestion with *BamH*I and *Not*I and ligation with T4 DNA ligase.
