# Supplementary material for: Investigation of Heterochromatin Protein 1 Function in the Malaria Parasite Plasmodium falciparum Using a Conditional Domain Deletion and Swapping Approach
Source: mSphere. 2021 Feb 3;6(1):e01220-20. doi: 10.1128/mSphere.01220-20 (PMC7860992; doi:10.1128/mSphere.01220-20)
Supplement: TABLE S1 [file mSphere.01220-20-st001.pdf]

TABLE S1. Oligonucleotides used in this study.

Application	Oligo name	Sequence (5'-3')
PCR for the cloning of transfection vectors	**R1**	tttaccatctccatttgtttcatttg
	**F2**	gttgaagaaacaaatattagaagaac
	**R3**	cagtgctagctgctgttctatatcttaatcttg
	**F4**	gaaacaaatggagatggtaaaggaataacctttgaacaattatatc
	**R5**	ctaatatttgtttcttcaacacctacattttcaaatactcgtac
	**F11**	cagtggatccaaaaaatgacaggtagtgatgaag
	**R12**	attcaaatgacctgttcttc
	**F35**	caaatgaaacaaatggagatggtaaaattatgaaaaaaaacaatgtacag
	**R36**	gttcttctaatatttgtttcttcaacccattgtggtgatgcattttttaag
	**F38**	gaagaacaggtcatttgaatataaaagttaacgattttaaaaaa
	**R41**	caatgcggccgcaaccgttctatatctaagtcttg
	**R42**	caatgcggccgctgctgttctatatcttaatcttg
	**F139**	gtaaataaaaaaaataatatacaataac
	**R143**	ctaaaagaatataaaatatataaatat
	**R148**	agggtatcaccttcaaacttgacttcagcacgtgtcttgtag
	**F158**	cgttggccgattcattaatgaaaggatattcagatgatgag
	**R159**	gttattgtatattatttttttatttacttacgctgttctatatcttaatc
	**F160**	atatttatatattttatattcttttagaaaggctattcagatgatg
	**R161**	gttcttctcctttactcataaccgttctatatctaagtc
	**F162**	atgagtaaaggagaagaac
	**R163**	cctcttcgctattacgccaggaggttaaaattctaaactatatg
	**F164**	atatttatatattttatattcttttagaaaggatatagtgatgatga
	**R165**	gttcttctcctttactcattgctgttctatatcttaatc
	**F177**	atatttatatattttatattcttttagaaaacaaatttcttatctaaag
	**R178**	gttcttctcctttactcatattcaaatgacctgttcttc
	**PCRA_F**	ctggcgtaatagcgaagagg
	**PCRA_R**	cattaatgaatcggccaacg
Diagnostic PCR on gDNA	**F119**	gtgtgtgtttaagaaaaaatatg
	**R157**	catgtagccaaaatatgtg
